# Influence of Residualizing Properties of the Radiolabel on Radionuclide Molecular Imaging of HER3 Using Affibody Molecules

**DOI:** 10.3390/ijms21041312

**Published:** 2020-02-15

**Authors:** Sara S. Rinne, Tianqi Xu, Charles Dahlsson Leitao, Stefan Ståhl, John Löfblom, Anna Orlova, Vladimir Tolmachev, Anzhelika Vorobyeva

**Affiliations:** 1 Department of Medicinal Chemistry, Uppsala University, 751 23 Uppsala, Sweden; sara.rinne@ilk.uu.se (S.S.R.); anna.orlova@ilk.uu.se (A.O.); 2 Department of Immunology, Genetics and Pathology, Uppsala University, 751 85 Uppsala, Sweden; tianqi.xu@igp.uu.se (T.X.); vladimir.tolmachev@igp.uu.se (V.T.); 3 Department of Protein Science, School of Engineering Sciences in Chemistry, Biotechnology and Health, KTH Royal Institute of Technology, 106 91 Stockholm, Sweden; chdl@kth.se (C.D.L.); ssta@kth.se (S.S.); lofblom@kth.se (J.L.); 4 Science for Life Laboratory, Uppsala University, 751 23 Uppsala, Sweden; 5 Centrum for Oncotheranostics, National Research Tomsk Polytechnic University, 634050 Tomsk, Russia

**Keywords:** HER3, affibody, radionuclide, molecular imaging, iodine, PIB

## Abstract

Human epidermal growth factor receptor type 3 (HER3) is an emerging therapeutic target in several malignancies. To select potential responders to HER3-targeted therapy, radionuclide molecular imaging of HER3 expression using affibody molecules could be performed. Due to physiological expression of HER3 in normal organs, high imaging contrast remains challenging. Due to slow internalization of affibody molecules by cancer cells, we hypothesized that labeling (HE)_3_-Z_HER3:08698_-DOTAGA affibody molecule with non-residualizing [^125^I]-*N*-succinimidyl-4-iodobenzoate (PIB) label would improve the tumor-to-normal organs ratios compared to previously reported residualizing radiometal labels. The [^125^I]I-PIB-(HE)_3_-Z_HER3:08698_-DOTAGA was compared side-by-side with [^111^In]In-(HE)_3_-Z_HER3:08698_-DOTAGA. Both conjugates demonstrated specific high-affinity binding to HER3-expressing BxPC-3 and DU145 cancer cells. Biodistribution in mice bearing BxPC-3 xenografts at 4 and 24 h pi showed faster clearance of the [^125^I]I-PIB label compared to the indium-111 label from most tissues, except blood. This resulted in higher tumor-to-organ ratios in HER3-expressing organs for [^125^I]I-PIB-(HE)_3_-Z_HER3:08698_-DOTAGA at 4 h, providing the tumor-to-liver ratio of 2.4 ± 0.3. The tumor uptake of both conjugates was specific, however, it was lower for the [^125^I]I-PIB label. In conclusion, the use of non-residualizing [^125^I]I-PIB label for HER3-targeting affibody molecule provided higher tumor-to-liver ratio than the indium-111 label, however, further improvement in tumor uptake and retention is needed.

## 1. Introduction

Human epidermal growth factor receptor type 3 (HER3) is a transmembrane protein belonging to the human epidermal growth factor receptor (HER) family, which is involved in the regulation of cellular proliferation, motility, and apoptosis [1]. The intracellular kinase domain of HER3 is impaired and depends on heterodimerization with other members of the HER family for signal transduction [2]. Heterodimerization with HER3 appreciably enhances the signaling of other HER receptors, of which the HER2-HER3 and HER1 (EGFR)-HER3 heterodimers cause potent mitogenic signaling [3,4]. Overexpression of HER3 has been documented in several malignancies, such as prostate [5], gastric [6], breast [7], pancreatic [8], lung [9], and ovarian [10] cancers. It has been suggested that overexpression of HER3 and HER3-mediated signaling are important molecular mechanisms in the resistance to anti-EGFR and anti-HER2 therapies [11,12,13]. Understanding the importance of HER3 signaling in cancer has spurred the development of HER3-targeting therapeutics [2,13,14]. Several types of HER3-targeting agents, such as HER3-blocking antibodies [15,16] and affibody molecules [17,18], as well as antibody-drug conjugates [19], are under active preclinical and clinical development. Monoclonal antibody seribantumab was studied in several clinical trials but did not prolong progression-free survival, however, no biomarkers were used for selection of patients enrolled for these trials [20]. These results suggest that the use of predictive biomarkers is necessary to select a sub-cohort of patients who would most likely benefit from HER3-targeted therapeutics [13,16]. At the same time, upregulation of HER3 in response to therapy suggests that biopsy sampling has to be performed repeatedly to detect onset of resistance. Naturally, such a repetitive and invasive procedure is hardly feasible in the clinics.

Radionuclide molecular imaging of HER3 expression levels using specific targeted probes appears to represent a more clinically relevant procedure [21]. This noninvasive test could be performed repeatedly with a minimal discomfort for the patient. Current approaches for development of HER3-imaging probes include the use of radiolabeled antibodies [22,23,24,25,26], antibody fragments [27], nanobody-based tracers [28], short peptides [29], and affibody molecules [30,31,32,33,34,35]. It has to be noted that development of HER3-imaging probes is challenging due to the pattern of HER3 expression. It is physiologically expressed in a number of adult tissues, for example in the liver and gastrointestinal tract [36]. Additionally, a moderate level of overexpression (less than 5 × 10^5^ receptors/cell) in malignant cells complicates obtaining a high contrast against background caused by uptake in normal tissues.

Our work on the development of HER3 imaging agents is focused on the use of affibody molecules as the targeting moiety. Affibody molecules are affinity proteins based on a three-helical domain from protein A [37]. A combination of high affinity and small size (58 amino acids, molecular weight of 7 kDa) makes affibody molecules an attractive type of imaging probe, providing high-contrast imaging within a few hours after injection [37,38]. Clinical studies have demonstrated that affibody-based high-contrast imaging of HER2-expressing tumors is possible at the day of injection [39].

Taking into account the ubiquitous expression of HER3, cross-reactive affibody molecules with equal affinity to human HER3 and the murine orthologue, mErbB3, have been selected for development of imaging probes [40]. In the case of HER3-targeting affibody molecules, high hepatic uptake appeared to be challenging [30,31,32]. High hepatic uptake prevents imaging of hepatic metastases, which are common in many cancers. It has been demonstrated that an adjustment of the injected protein dose permits partial saturation of mErbB3 in the liver without reducing the uptake of an anti-HER3 [^68^Ga]Ga-HEHEHE-Z_08698_-NOTA affibody molecule in tumors [31]. Nonetheless, liver uptake still exceeded the tumor uptake. Based on this, it was suggested that the hepatic uptake of anti-HER3 affibody molecules is determined by two different mechanisms; one is mErbB3/HER3-mediated and another is dependent on off-target interactions of affibody molecules. Further studies showed that the off-target interactions can be modulated by the composition of the radionuclide-chelator complex [31,33,34,35]. The best tumor-to-liver ratio of 2.3 ± 0.5 was obtained at 24 h post-injection (pi) by a co-injection of [^57^Co]Co-NOTA-Z_HER3:08699_ with a three-fold molar excess of unlabeled (Z08699)_3_ HER3-affibody trimer [41].

The goal of this study was to evaluate an alternative approach for improving tumor-to-liver ratios for anti-HER3 affibody molecules. Slow internalization after binding to receptors on cancer cells is a common feature of many engineered scaffold proteins (ESPs), such as affibody molecules [33,34,35], ADAPTs [42], and DARPins [43,44]. Due to the slow internalization, good retention of activity is more dependent on high affinity of the targeting protein and to a lesser extent on the residualizing properties of the radiolabel. In contrast, internalization after uptake of scaffold proteins in excretory organs is typically rapid, and the use of a non-residualizing radioiodine label results in a rapid leakage of activity from these organs. This has been used to improve tumor-to-organ ratios for ESPs, e.g., tumor-to-liver [45] and tumor-to-kidney [46] ratios for affibody molecules, tumor-to-kidney ratio for ADAPTs [42], as well as tumor-to-kidney and tumor-to-liver ratios for DARPins [43,44]. It should be noted that this approach was applied for ESPs targeting HER2, which has a high level of receptor overexpression in vitro (e.g., 1.6 × 10^6^ receptors/cell in SKOV-3 cells [47]) and in tumor models in vivo. In this study, we intended to test the hypothesis that the use of a non-residualizing radioiodine label would improve the tumor-to-liver ratio for the HER3-targeting Z_HER3:08698_ affibody molecule compared to a residualizing radiometal label.

To test this hypothesis, we used a (HE)_3_-Z_HER3:08698_-DOTAGA affibody molecule, as this affibody molecule with the (HE)_3_-tag demonstrated the best tumor-to-liver ratio with an indium-111 label in the previous study [33]. Labeling it with indium-111 provided [^111^In]In-(HE)_3_-Z_HER3:08698_-DOTAGA, which was used as a control having residualizing label properties. To obtain a non-residualizing label, the same affibody molecule was used for indirect radioiodination using [^125^I]-*N*-succinimidyl-4-iodobenzoate. This labeling approach was selected because the radiometabolites of [^125^I]-4-iodobenzoate label are rapidly excreted and do no contribute to background [44,48]. Iodine-125 was used in this study for radioiodination. Due to long half-life and low-energy gamma emission, this nuclide is not suitable for imaging in humans. However, this nuclide is a convenient surrogate for ^123^I and ^124^I, which can be used for clinical SPECT and PET imaging, respectively. The DOTAGA chelator was not used for radiolabeling in this case but was utilized as a biodistribution modifier because an increased negative charge at the C-terminus reduces the hepatic uptake of Z_HER3:08698_ [33]. The (HE)_3_-tag at *N*-terminus was also used to reduce unspecific hepatic uptake [34].

## 2. Results

### 2.1. Radiolabeling and Stability

Indirect radioiodination of (HE)_3_-Z_HER3:08698_-DOTAGA using [^125^I]I-PIB linker [49] was performed in 19% ± 2% (*n* = 3) radiochemical yield. Specific activity of 1.2 MBq/µg (10.1 MBq/nmol) was achieved. Purification using NAP-5 column provided the radiolabeled affibody molecule with 99% radiochemical purity. Isolated yield after purification was 18% ± 1% (*n* = 3). No release of activity during incubation with an excess of KI or in 30% ethanol was observed (Table 1).

Radiolabeling of (HE)_3_-Z_HER3:08698_-DOTAGA with indium-111 [33] was initially performed in 38% radiochemical yield. Addition of acetonitrile to the reaction mixture lead to an increase in the yield up to 65%. Specific activity of 1.0 MBq/µg (8.1 MBq/nmol) was achieved. Purification using size-exclusion columns provided the radiolabeled affibody molecule with 99% radiochemical purity. Isolated yield after purification was 45% ± 11% (*n* = 2). No release of activity during EDTA challenge was observed (Table 1).

### 2.2. In Vitro Studies

**Figure 1:**
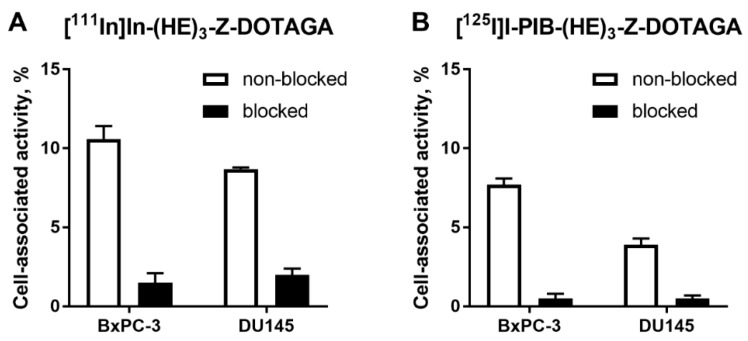
Binding specificity of (**A**) [^111^In]In-(HE)_3_-Z_HER3:08698_-DOTAGA and (**B**) [^125^I]I-PIB-(HE)_3_-Z_HER3:08698_-DOTAGA to human epidermal growth factor receptor type 3 (HER3)-expressing BxPC-3 and DU145 cancer cells in vitro. Concentration of radiolabeled compounds was 0.05 nM; for blocking, 1000-fold molar excess of non-labeled affibody molecule was added. Data are presented as mean from three samples ± standard deviation (SD).

In vitro evaluation was performed using BxPC-3 (pancreatic cancer) and DU145 (prostate cancer) cells according to Wållberg and Orlova [50]. To demonstrate binding specificity of radiolabeled affibody molecules to HER3, the HER3 receptors were saturated with 1000-fold molar excess of a non-labeled HER3-targeting affibody molecule before addition of the radiolabeled conjugates. Blocking of the HER3 receptors resulted in a significant (*p* < 0.001, *t*-test) decrease of uptake of the radiolabeled molecules. This showed that both radiolabeled conjugates retained the HER3-specific binding after the labeling (Figure 1). The uptake in BxPC-3 cells was higher than in DU145 cells.

The binding kinetics of [^125^I]I-PIB-(HE)_3_-Z_HER3:08698_-DOTAGA and [^111^In]In-(HE)_3_-Z_HER3:08698_-DOTAGA to BxPC-3 cells were measured using LigandTracer [51]. The *K*_D_ values for both conjugates were in the picomolar range (Table 2), with the indium-labeled conjugate having higher affinity than the radioiodinated one.

**Figure 2:**
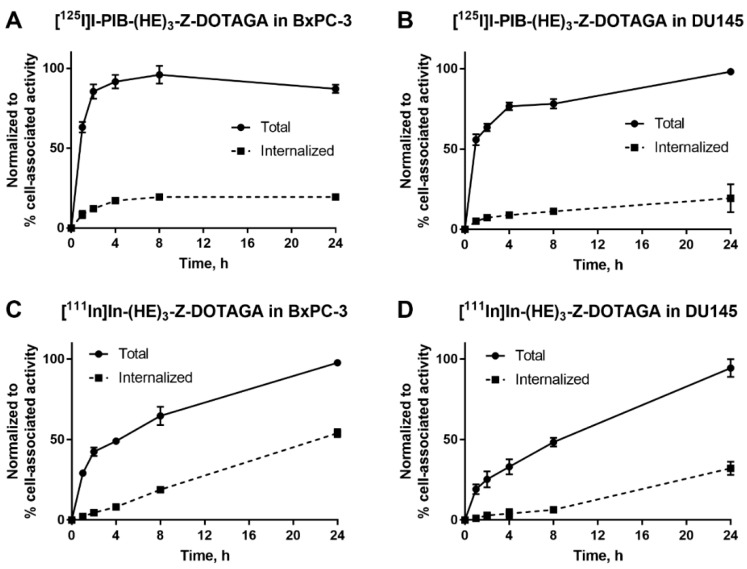
Cellular processing of (**A**,**B**) [^125^I]I-PIB-(HE)_3_-Z_HER3:08698_-DOTAGA and (**C**,**D**) [^111^In]In-(HE)_3_-Z_HER3:08698_-DOTAGA by HER3-expressing BxPC-3 and DU145 cells during continuous incubation over 24 h. Data are presented as mean from three samples ± SD; error bars may not be visible when they are smaller than symbols.

The processing and internalization of radiolabeled affibody molecules by the HER3-expressing cells during continuous incubation is shown in Figure 2. For the radioiodinated conjugate, rapid association was followed by a plateau, while for the radiometal-labeled conjugate, an increase of cell-associated activity was observed over 24 h. The internalized fractions at 24 h were lower for affibody molecules labeled with non-residualizing iodine-125 label in comparison to residualizing indium-111 label. After 4 h of incubation, the internalized fraction for the indium-111 label was below 20% from total cell-associated activity in both cell lines. By 24 h, ca. 50% of cell-associated activity was internalized in BxPC-3 cells and ca. 30% was internalized in DU145 cells.

### 2.3. In Vivo Studies

**Figure 3:**
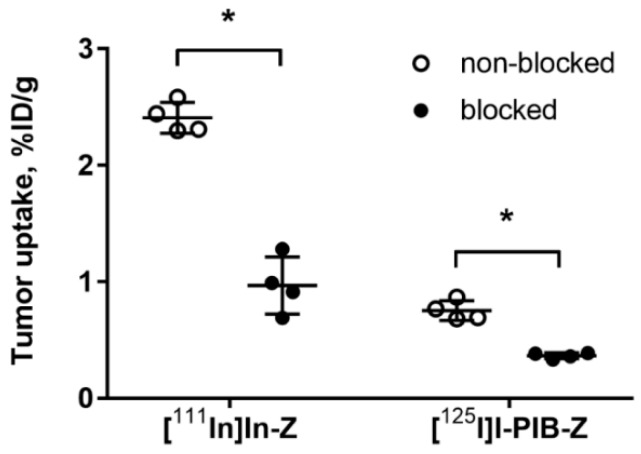
Specificity of [^125^I]I-PIB-(HE)_3_-Z_HER3:08698_-DOTAGA and [^111^In]In-(HE)_3_-Z_HER3:08698_-DOTAGA tumor targeting in Balb/c nu/nu mice bearing BxPC-3 xenografts. The uptake of both imaging probes in tumors was significantly (* *p* < 0.0001) decreased when a large excess of non-labeled HER3 affibody molecule was administered. Data are presented as average ± SD for four mice.

Both radiolabeled conjugates demonstrated specific binding in vivo to HER3-expressing BxPC-3 xenografts (Figure 3). Uptake of activity in BxPC-3 xenografts was significantly (*p* < 0.0001) reduced when 72 µg of HER3 affibody molecule was injected compared to 2 µg.

Biodistribution of [^125^I]I-PIB-(HE)_3_-Z_HER3:08698_-DOTAGA and [^111^In]In-(HE)_3_-Z_HER3:08698_-DOTAGA was studied in Balb/c nu/nu mice bearing BxPC-3 xenografts at 4 and 24 h pi (Table 3). Both radiolabeled conjugates showed biodistribution characteristic for affibody molecules with rapid and predominantly renal clearance. However, there were a number of differences between the radioiodine- and the radiometal-labeled conjugates. The uptake of the radioiodine-labeled conjugate was generally several fold lower in normal organs and tissues, except blood. Especially prominent were the differences in kidney uptake, 291 ± 39%ID/g for the indium label vs. 2.7 ± 0.7%ID/g for the radioiodine label at 4 h pi, which further reduced to 0.15 ± 0.02%ID/g by 24 h. The tumor uptake was lower for the radioiodine label as well, 0.8 ± 0.1%ID/g vs. 2.4 ± 0.1%ID/g for the indium label at 4 h pi. The indium label also demonstrated better retention in the tumor than radioiodine at 24 h.

Due to lower retention in the blood and higher retention in the tumor, the radiometal label provided significantly (*p* < 0.05) higher tumor-to-blood, tumor-to-lung, and tumor-to-bone ratios than the radioiodine label at 4 h (Table 4). Tumor-to-spleen and tumor-to-muscle ratios were comparable between the labels. On the other hand, the radioiodine label provided higher tumor-to-organ ratios in HER3-expressing organs (salivary gland, stomach, small intestine, and liver) at 4 h (Table 4). By 24 h there was no marked increase in tumor-to-organ ratios for both labels, except the tumor-to-blood ratio for the ^111^In-labeled conjugate.

### 2.4. Imaging

**Figure 4:**
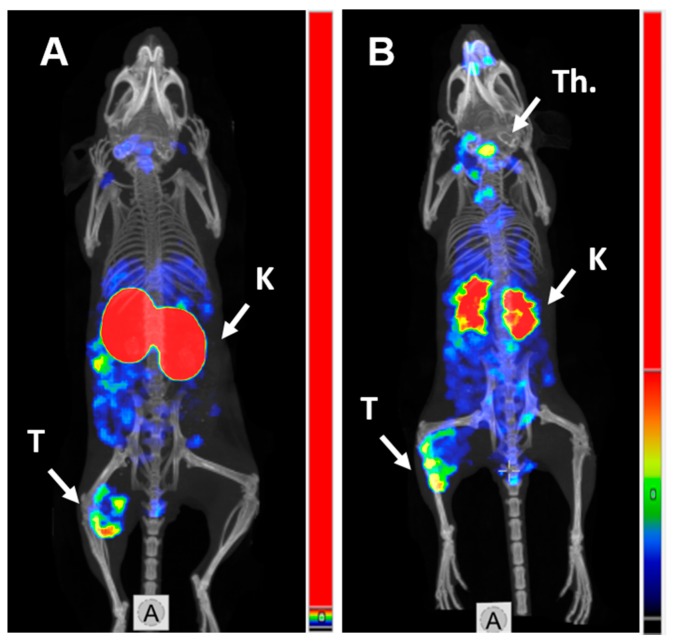
MicroSPECT/CT imaging of HER3 expression in BxPC3 xenografts in Balb/c nu/nu mice using (**A**) [^111^In]In-(HE)_3_-Z_HER3:08698_-DOTAGA and (**B**) [^125^I]I-PIB-(HE)_3_-Z_HER3:08698_-DOTAGA 4 h pi. K—kidneys; T—tumor; Th.—thyroid.

SPECT/CT imaging in Balb/c nu/nu mice at 4 h pi using [^125^I]I-PIB-(HE)_3_-Z_HER3:08698_-DOTAGA and [^111^In]In-(HE)_3_-Z_HER3:08698_-DOTAGA confirmed the results of biodistribution studies (Figure 4). Both radiolabeled conjugates provided clear visualization of HER3-expressing BxPC3 xenografts. Non-residualizing radioiodine label demonstrated lower retention of activity in kidneys compared to the radiometal label.

## 3. Discussion

The use of a novel class of imaging probes based on engineered scaffold proteins is approaching clinical practice [52]. Despite promising characteristics, only a few groups work with engineered scaffold proteins, and the available information concerning influence of different factors on their imaging properties is scarce. Particularly challenging is the development of probes for imaging of targets that are expressed not only in tumors but also in normal tissues, such as EGFR, IGF-1R, or HER3. However, the data suggest that an optimal molecular design and selection of an optimal labeling approach may noticeably enhance imaging contrast resulting in improved sensitivity. One of the substantial challenges in radionuclide molecular imaging of HER3 expression in tumors is to increase the tumor-to-liver ratio. Earlier, we investigated possibilities to minimize uptake of HER3 imaging probes in the liver. Our approach was based on the combination of dose adjustment (to minimize specific uptake) and optimization of molecular design, including selection of an optimal nuclide/chelator combination (to minimize unspecific uptake). This approach increased the tumor-to-liver ratio by ca. 7-fold for EGFR-targeting affibody molecules from 0.46 for [^111^In]In-DOTA-Z_2377_ (4 h pi) [53] to 3.1 for [^57^Co]Co-DOTA-Z_2377_ (3 h pi) [54] in an A431 xenograft model. For HER3, the effect was less impressive with the tumor-to-liver ratio increasing by ca. 4-fold from 0.6 for [^68^Ga]-(HE)_3_-Z_HER3:08698_ -NOTA (3 h pi) [31] to 2.3 for [^57^Co]Co-NOTA-Z_HER3:08698_ (24 h pi, co-injected with (Z_HER3:08698_)_3_ trimer) [41] in a BxPC-3 xenograft model. It has to be noted that although both EGFR and HER3 are expressed in normal tissues, including liver, the expression level of HER3 in tumors is appreciably lower than the level of EGFR. In this study, we evaluated an alternative strategy aiming towards decreasing the retention of the radionuclide in the liver rather than reducing its uptake. To achieve this, we included two modifications in the molecular design of a new tracer, that were previously shown to decrease hepatic uptake, i.e., (HE)_3_-tag at the *N*-terminus and DOTAGA chelator at the C-terminus.

The use of [^125^I]*N*-succinimidyl-4-iodobenzoate resulted in a stable labeling of (HE)_3_-Z_HER3:08698_-DOTAGA (Table 1). In vitro binding of [^125^I]I-PIB-(HE)_3_-Z_HER3:08698_ -DOTAGA to HER3-expressing cell lines was specific as demonstrated in a saturation experiment (Figure 1). The affinity of the radioiodinated variant was lower compared to the ^111^In-labeled counterpart (Table 2), but remained high, 98 ± 12 pM, which was considered as a precondition for successful in vivo targeting. The results of in vitro evaluation of cellular processing were somewhat surprising, as the internalization of [^111^In]In-(HE)_3_-Z_HER3:08698_ -DOTAGA was somewhat faster (54% at 24 h for BxPC-3 cells) compared with the earlier reported internalization of [^111^In]In-Z_HER3:08698_ -DOTAGA without (HE)_3_-tag (around 30% at 24 h for BxPC-3 cells) [33]. However, only 18% of [^111^In]In-(HE)_3_-Z_HER3:08698_ -DOTAGA was internalized at 4 h, which indicated that the use of a non-residualizing label would not be associated with a major loss of cell-associated activity within a few hours after injection. Although the general pattern of cellular processing of [^111^In]In-(HE)_3_-Z_HER3:08698_-DOTAGA was similar for BxPC-3 and DU-145 cells, the internalization rate was somewhat lower for DU-145 cells. This correlated with the pattern of the [^125^I]I-PIB-(HE)_3_-Z_HER3:08698_-DOTAGA processing. There was a slight increase of the cell-associated activity in DU-145 cells between 4 and 24 h, while the cell-associated activity in BxPC-3 cells reached a plateau at 4 h with a tendency for a decrease. This could indicate a faster release of [^125^I]I-PIB-(HE)_3_- Z_HER3:08698_-DOTAGA catabolites from BxPC-3 cells due to a more rapid internalization.

The results of an in vivo saturation test (Figure 3) demonstrated that the tumor uptake of both [^125^I]I-PIB-(HE)_3_-Z_HER3:08698_-DOTAGA and [^111^In]In-(HE)_3_-Z_HER3:08698_-DOTAGA was significantly reduced by saturating HER3 receptors by co-injection of a large excess of non-labeled Z_HER3:08698_. This confirmed that the tumor uptake of both variants was HER3-specific.

The distribution of activity for [^125^I]I-PIB-(HE)_3_-Z_HER3:08698_-DOTAGA and [^111^In]In-(HE)_3_- Z_HER3:08698_-DOTAGA was obviously different (Table 3). Already at 4 h pi the activity of ^125^I in the majority of normal tissues was substantially lower compared to ^111^In. The most striking difference was the more than 100-fold reduction of renal activity. The reduction of activity in other HER3-expressing tissues (salivary gland, liver, stomach, small intestines) was 6–10-fold for the non-residualizing [^125^I]I-PIB label. Decrease of the liver uptake was approximately 10-fold. The level of blood-born activity was higher for the radioiodinated variant. This is most likely caused by released radioiodine metabolites from kidneys and liver while the radiometabolites of ^111^In-label were trapped inside tissues where [^111^In]In-(HE)_3_-Z_HER3:08698_-DOTAGA was taken up. Earlier, we have shown for another scaffold protein with high renal uptake, ADAPT, that the rapid decrease of activity in kidneys for a non-residualizing radioiodine label is associated with higher percentage of low-molecular-weight fraction in blood in comparison with a radiometal label [42]. The tumor uptake was also lower for [^125^I]I-PIB-(HE)_3_-Z_HER3:08698_-DOTAGA, but the decrease was 3.5-fold, i.e., smaller compared to decrease for normal tissues. Thus, the ratios of uptake in tumors to uptake in majority of tissues were higher for [^125^I]I-PIB-(HE)_3_-Z_HER3:08698_-DOTAGA compared to [^111^In]In-(HE)_3_-Z_HER3:08698_-DOTAGA at 4 h pi (Table 4). The exceptions were tumor-to-blood and tumor-to-bone ratios. A SPECT/CT image (Figure 4) confirmed the biodistribution data. At 24 h after injection, there was a substantial reduction of tumor uptake of [^125^I]I-PIB-(HE)_3_-Z_HER3:08698_-DOTAGA compared to 4 h, which reflects the increasing internalization of the tracer and leakage of radiometabolites. It has to be noted that the increase of tumor-to-organ ratios for [^125^I]I-PIB-(HE)_3_-Z_HER3:08698_-DOTAGA at 4 h pi was obtained at the cost of noticeable decrease of tumor uptake. The decrease in tumor uptake was more substantial than it could be expected from in vitro cellular processing data. As cells in vivo are influenced by a variety of signaling substances, such influence is difficult to mimic in vitro (if possible at all). The increased internalization rate of HER3 or HER3/[^125^I]I-PIB-(HE)_3_-Z_HER3:08698_-DOTAGA adduct might be a consequence of such influence. This suggests that in vitro modeling would not be quite predictive for in vivo situation. Nevertheless, the tumor-to-liver ratio of 2.4 ± 0.3 was achieved for [^125^I]I-PIB-(HE)_3_-Z_HER3:08698_-DOTAGA, which is one of the best for anti-HER3 affibody molecules. Overall, the results of this study suggest that the hypothesis that tumor-to-liver and other tumor-to-organ ratios can be improved by the use of a non-residualizing label is correct.

While this study demonstrated feasibility of the use of non-residualizing labels for enhancing the tumor-to-liver ratio of anti-HER3 affibody molecule, the low tumor uptake is of concern. Further strategies for development of this approach could include modulation of radiometabolite retention and increase of affinity. [^125^I]I-para-iodobenzoate is only one of the potential pendant groups providing a non-residualizing radioiodine label. Alternatively, labeled iodo-hydroxyphenylethyl-maleimide (I-HPEM) [55,56] or 4-iodophenethylmaleimide (I-PEM) [57] can be used for indirect radioiodination of affibody molecules. These alternative pendant groups have different rates of excretion from normal tissues compared to PIB, which opens an opportunity for finding the most suitable variant. Non-residualizing properties are also typical for radiofluorine labels [58,59,60]. Importantly, all these labels are site-specific, and their use might help to avoid undesirable lysine modifications close to the binding site of the affibody molecule and might increase the binding affinity to HER3. This might also increase the tumor retention of activity. Furthermore, although (HE)_3_-tag reduced non-specific hepatic uptake of affibody molecules, the presence of (HE)_3_-tag accelerated the internalization rates. Thus, the use of an affibody without (HE)_3_-tag should be evaluated in combination with non-residualizing labels.

The use of murine model in this feasibility study was based on an assumption that rates of internalization of affibody molecules by human and murine hepatocytes are similar. However, the rates might be different in rodents and humans. A dual-label microdosing clinical study would be necessary for confirmation of translational potential of our findings.

Although outside the scope of this study, it is worth mentioning that [^111^In]In-(HE)_3_-Z_HER3:08698_-DOTAGA demonstrated an overall lower uptake and retention in normal organs (ca. eight times lower in blood, two times lower in lungs and liver at 4 h pi) in comparison with the previously reported [^111^In]In-Z_HER3:08698_-DOTAGA HER3 affibody molecule without the (HE)_3_-tag [33]. Even though the tumor uptake of the (HE)_3_-tagged variant was lower (2.4 ± 0.1%ID/g vs. 3.2 ± 0.1%ID/g), the tumor-to-organ ratios were higher. Moreover, [^111^In]In-(HE)_3_-Z_HER3:08698_-DOTAGA provided higher tumor-to-organ ratios than any ^111^In-labeled Z_HER3:08698_ variant tested before [33]. For example, the tumor-to-blood ratio of 88 ± 9 (24 h pi) was more than four-fold higher than any value obtained previously for any Z_HER3:08698_ derivative. These data emphasize the strength of molecular design optimization.

## 4. Materials and Methods

### 4.1. General Materials and Instruments

Indium chloride [^111^In]InCl_3_ was purchased from Mallinckrodt Pharmaceuticals (Staines-upon-Thames, UK). Sodium iodide [^125^I]NaI was purchased from Perkin Elmer Sverige AB (Hägersten, Sweden). Instant thin-layer chromatography (iTLC) analysis was performed using iTLC silica gel strips (Varian, Lake Forest, CA, USA). The activity distribution was measured using a Cyclone storage phosphor system (Packard) and analyzed by OptiQuant image analysis software (Perkin Elmer, Waltham, MA, USA). Purification was performed using NAP-5 columns (GE Healthcare, Little Chalfont, United Kingdom) pre-equilibrated with 1% BSA in PBS and eluted with PBS. Activity was measured using an automated gamma-spectrometer with a NaI(TI) detector (1480 Wizard, Wallac, Finland). BxPC-3 and DU145 cells were purchased from the American Type Culture Collection (ATCC) and were cultured in RPMI medium supplemented with 10% fetal bovine serum (FBS), 2 mM L-glutamine, 100 IU/mL penicillin, and 100 µg/mL streptomycin in a humidified incubator with 5% CO_2_ at 37 °C, unless stated otherwise.

### 4.2. Protein Production

The HER3-binding Z_HER3:08698_ affibody bearing HEHEHE-sequence at *N*-terminus and the DOTAGA chelator at C-terminus, denoted as (HE)_3_-Z_HER3:08698_-DOTAGA, was produced, purified, and characterized as described earlier [35].

### 4.3. Radiolabeling and Label Stability

Indirect radioiodination of (HE)_3_-Z_HER3:08698_-DOTAGA using *N*-succinimidyl-para-(trimethylstannyl)benzoate was performed as described earlier for affibody molecules [49] and DARPins [48]. An aqueous solution of 0.1% acetic acid (60–90 µL) was added to radioiodine (40–45 µL, 135–180 MBq). Then, *N*-succinimidyl-p-(trimethylstannyl)benzoate (6.5 nmoles, 2.5 µg, 2.5 µL of 1 mg/mL in 5% acetic acid in methanol) and chloramine-T (80 µg, 10 µL, 8 mg/mL in water) were added. After incubation at room temperature for 5 min, the reaction was stopped by addition of sodium metabisulfite (120 µg, 10 µL, 12 mg/mL in water). Then, (HE)_3_-Z_HER3:08698_-DOTAGA (3.7 nmoles, 30 µg, 15 µL of 2 mg/mL in water) in 100–150 µL of 0.07 M borate buffer (pH 9.3) was added. After 30 min of incubation at room temperature, the radiolabeled conjugate was purified on a NAP-5 column. The labeling yield was determined by radio-iTLC analysis in 4:1 acetone:water system.

For labeling with indium-111, (HE)_3_-Z_HER3:08698_-DOTAGA (20 µg in 10 µL water) was added to 20 µL of acetonitrile and incubated with 30 µL of [^111^In]InCl_3_ (29 MBq) for 90 min at 85 °C. To remove any unbound activity, an intermediate challenge with 500-fold excess of EDTA for 10 min at 85 °C was performed before purification on a NAP-5 column. The labeling yield was determined by radio-iTLC analysis in 0.2 M citric acid.

The in vitro stability test was performed by incubating [^125^I]I-PIB-(HE)_3_-Z_HER3:08698_ -DOTAGA with 5000-fold molar excess of KI in PBS and by incubating it in 30% ethanol at room temperature for 4 h. The stability of [^111^In]In-labeled affibody molecule was tested by incubating it with 5000-fold excess of EDTA at room temperature for 4 h. Control samples were incubated in PBS, analysis was done using radio-iTLC.

### 4.4. Binding Specificity and Cellular Processing Assays

In vitro studies were performed using HER3-expressing cancer cell lines BxPC-3 (12 ± 2 × 10^3^ receptors/cell) [31] and DU145 (estimated as 60% of the expression level in BxPC-3 cells). Cells were seeded in 3 cm petri dishes (ca. 10^6^ cells per dish), a set of three dishes was used for each group.

Binding specificity to HER3 was evaluated as described previously [50]. To saturate HER3 receptors, 1000-fold excess of non-labeled anti-HER3 affibody molecule (50 nM) was added to one group of cells, media was only added to the second group. After 15 min incubation at room temperature, radiolabeled [^125^I]I-PIB-(HE)_3_-Z_HER3:08698_-DOTAGA or [^111^In]In-(HE)_3_-Z_HER3:08698_ -DOTAGA was added to both groups at 0.05 nM final concentration. After incubation for 1 h at 37 °C, the cell medium was collected, cells were washed with 1 mL of medium, and 1 mL of 1 M NaOH was added to lyse the cells. After 30 min of incubation, the cell lysate was collected. The radioactivity in each fraction was measured to calculate the percent of cell-bound radioactivity.

Cellular retention and processing of radiolabeled affibody molecules was studied during continuous incubation by an acid-wash method [50]. Radiolabeled affibody molecules (0.1 nM) were added to cells and incubated at 37 °C in a humidified incubator for 1, 2, 4, 8, and 24 h. At the selected time point, the media was collected from one set of dishes and cells were washed once with serum-free media (1 mL). To collect the membrane-bound fraction, the cells were treated with 0.2 M glycine buffer containing 4 M urea, pH 2.0 (1 mL) on ice for 5 min, the buffer was collected, and the cells were washed once with the same buffer (1 mL). To collect the internalized fraction, the cells were treated with 1 M NaOH (1 mL) for 30 min, the cells were collected and additionally washed with 1 mL. The activity in every fraction was measured and the percentage of cell-associated activity was calculated. Every dataset in Figure 2 represents the processing of one radiolabeled conjugate in one cell line. The maximum value of cell-associated activity in each dataset (at 8 or 24 h) was taken as 100% and the other dataset values were normalized to it.

### 4.5. Affinity Measurements Using LigandTracer

The binding kinetics of radiolabeled affibody molecules to living BxPC-3 cells was measured using LigandTracer and evaluated using the TraceDrawer Software (all from Ridgeview Instruments, Vänge, Sweden) as described earlier [51]. Increasing concentrations of radiolabeled affibody molecules (1 and 5 nM) were added to cells, followed by the change of media and measurements of retention in the dissociation phase. Kinetics was recorded at room temperature and dissociation constants were calculated based on association and dissociation rates.

### 4.6. Animal Studies

The animal experiments were planned and performed according to national legislation on protection of laboratory animals. The animal studies were approved by the local ethics committee for animal research in Uppsala, Sweden (ethical permission C5/16 from 26-02-2016).

For implantation of tumors, 5 × 10^6^ of BxPC-3 cells in 100 µL of media were subcutaneously injected in the right hind leg of female Balb/c nu/nu mice. The experiments were performed three weeks after implantation. The average animal weight was 17 ± 1 g. The average tumor weight was 0.10 ± 0.06 g. Comparative biodistribution of [^125^I]I-PIB-(HE)_3_-Z_HER3:08698_ -DOTAGA and [^111^In]In-(HE)_3_- Z_HER3:08698_-DOTAGA was studied using a dual-label approach. The mice were intravenously (i.v.) injected with a mixture of [^125^I]I-PIB-(HE)_3_-Z_HER3:08698_-DOTAGA and [^111^In]In-(HE)_3_-Z_HER3:08698_-DOTAGA in 100 μL of 1% bovine serum albumin in PBS per mouse (total protein dose 2 µg, 15 kBq for [^125^I]I label, 20 kBq for [^111^In]In label). To demonstrate the HER3-mediated uptake, the protein dose was increased to 72 µg using non-labeled anti-HER3 affibody molecule in one group of mice. At 4 and 24 h post-injection (pi) mice were anesthetized by an intraperitoneal injection of ketamine and xylazine solution and sacrificed by heart puncture. The dose of ketamine was 250 mg/kg, and the dose of xylazine was 25 mg/kg. The organs and tissues were collected, weighed, and the activity was measured using an automated γ-spectrometer. The percentage of injected dose per gram of sample (%ID/g) was calculated. Data for the intestines with contents and carcass were calculated as %ID per whole sample.

Whole body SPECT/CT scans of mice bearing BxPC-3 tumors and injected with [^125^I]I-PIB-(HE)_3_- Z_HER3:08698_-DOTAGA (2 μg, 1.9 MBq) or with [^111^In]In-(HE)_3_-Z_HER3:08698_-DOTAGA (2 μg, 1.5 MBq) were performed using nanoScan SPECT/CT (Mediso Medical Imaging Systems, Hungary). Imaging at 4 and 24 h pi was performed after mice were sacrificed by CO_2_ and urinary bladders were removed. The acquisition time was 20 min. In the case of ^125^I, energy window between 26 and 31 keV was used. For ^111^In, gamma-peaks of 245 and 171 keV (window width of 20%) were used for acquisition. CT scans were acquired using the following parameters: X-ray energy peak of 50 keV; 670 µA; 480 projections; and 5.26 min acquisition time. SPECT raw data were reconstructed using Tera-Tomo™ 3D SPECT reconstruction technology (version 3.00.020.000; Mediso Medical Imaging Systems Ltd., Budapest, Hungary): normal dynamic range; 30 iterations; and one subset. CT data were reconstructed using Filter Back Projection in Nucline 2.03 Software (Mediso Medical Imaging Systems Ltd., Budapest, Hungary). SPECT and CT files were fused using Nucline 2.03 Software (Mediso Medical Imaging Systems Ltd., Budapest, Hungary) and are presented as maximum intensity projections in the RGB color scale.

### 4.7. Statistical Analysis of the Data

Statistical analysis was performed using GraphPad Prism (version 7.02; GraphPad Software, Inc., La Jolla, CA, USA). *p* < 0.05 was considered a statistically significant difference. The in vitro data were analyzed using an unpaired two-tailed *t*-test. A paired two-tailed *t*-test was applied for the analysis of biodistribution data from the dual-label study to find significant differences.

## 5. Conclusions

In conclusion, this study demonstrated the feasibility of increasing tumor-to-liver contrast for HER3-targeting affibody molecules by the use of a non-residualizing label. However, further studies are required to provide better uptake in tumors.

## Figures and Tables

**Table 1:** In vitro stability of [^125^I]I-PIB-(HE)_3_-Z_HER3:08698_-DOTAGA and [^111^In]In-(HE)_3_-Z_HER3:08698_-DOTAGA. Radioiodinated affibody molecule was incubated for 4 h with 5000-fold molar excess of KI in PBS or with 30% ethanol and compared to PBS control. Indium-labeled affibody molecule was incubated with 5000-fold molar excess of EDTA. Analysis was performed in duplicates.

In Vitro Stability Test	[^111^In]In-(HE)_3_-Z_HER3:08698_-DOTAGA		[^125^I]I-PIB-(HE)_3_-Z_HER3:08698_-DOTAGA		
	5000× EDTA	PBS	5000× KI	30% EtOH	PBS
Protein-associated activity, %	99 ± 0	99 ± 0	98 ± 0	99 ± 0	98 ± 0

**Table 2:** Equilibrium dissociation constants for the interaction between radiolabeled affibody molecules and HER3-expressing BxPC-3 cells.

Compound	*K_D1_* (pM)
[^125^I]I-PIB-(HE)_3_-Z_HER3:08698_-DOTAGA	98 ± 12
[^111^In]In-(HE)_3_-Z_HER3:08698_-DOTAGA	19 ± 1

**Table 3:** Biodistribution of [^125^I]I-PIB-(HE)_3_-Z_HER3:08698_-DOTAGA and [^111^In]In-(HE)_3_-Z_HER3:08698_-DOTAGA in Balb/c nu/nu mice bearing BxPC3 xenografts at 4 and 24 h pi. Uptake is presented as percent of injected dose per gram (%ID/g). Results are presented as average ± SD of four mice.

Organ	4 h		24 h	
	[^125^I]I-PIB	[^111^In]In	[^125^I]I-PIB	[^111^In]In
Blood	0.37 ± 0.05 ^a,b^	0.06 ± 0.01 ^c^	0.05 ± 0.01	0.019 ± 0.002
Salivary glands	0.18 ± 0.01 ^a,b^	1.1 ± 0.1 ^c^	0.02 ± 0.01	0.78 ± 0.07
Lung	0.49 ± 0.06 ^a,b^	0.9 ± 0.2 ^c^	0.04 ± 0.01	0.31 ± 0.03
Liver	0.32 ± 0.04 ^a,b^	3.3 ± 0.6 ^c^	0.05 ± 0.01	2.1 ± 0.4
Spleen	0.12 ± 0.01 ^a,b^	0.5 ± 0.2 ^c^	0.027 ± 0.004	0.21 ± 0.02
Stomach	0.22 ± 0.04 ^a,b^	1.3 ± 0.1 ^c^	0.02 ± 0.01	0.7 ± 0.1
Small intestine	0.30 ± 0.07 ^a,b^	3.7 ± 0.8 ^c^	0.02 ± 0.02	1.6 ± 0.2
Kidney	2.7 ± 0.7 ^a,b^	291 ± 39 ^c^	0.15 ± 0.02	211 ± 28
Tumor	0.8 ± 0.1 ^a,b^	2.4 ± 0.1 ^c^	0.06 ± 0.04	1.7 ± 0.1
Muscle	0.05 ± 0.02 ^a,b^	0.14 ± 0.02 ^c^	0.008 ± 0.002	0.07 ± 0.01
Bone	0.4 ± 0.3	0.3 ± 0.1 ^c^	0.07 ± 0.02	0.10 ± 0.02

Differences were significant (*p* < 0.05) between: ^a^ the uptake of (HE)_3_-Z_HER3:08698_-DOTAGA labeled with [^125^I]I-PIB and [^111^In]In at the same time point (paired *t*-test); ^b^ the uptake of [^125^I]I-PIB-(HE)_3_-Z_HER3:08698_-DOTAGA at 4 and 24 h (unpaired *t*-test); ^c^ the uptake of [^111^In]In-(HE)_3_-Z_HER3:08698_-DOTAGA at 4 and 24 h (unpaired *t*-test).

**Table 4:** Tumor-to-organ ratios for [^125^I]I-PIB-(HE)_3_-Z_HER3:08698_-DOTAGA and [^111^In]In-(HE)_3_-Z_HER3:08698_-DOTAGA in Balb/c nu/nu mice bearing BxPC3 xenografts at 4 and 24 h pi. Results are presented as average ± SD of four mice.

Organ	4 h		24 h	
	[^125^I]I-PIB	[^111^In]In	[^125^I]I-PIB	[^111^In]In
Blood	2.0 ± 0.2 ^a,b^	43 ± 4 ^c^	1.6 ± 0.2 ^a^	88 ± 9
Salivary glands	4.1 ± 0.4 ^a,b^	2.2 ± 0.1	3.3 ± 0.2 ^a^	2.1 ± 0.1
Lung	1.5 ± 0.2 ^a^	2.6 ± 0.3 ^c^	1.7 ± 0.3 ^a^	5.4 ± 0.7
Liver	2.4 ± 0.3 ^a,b^	0.7 ± 0.1	1.6 ± 0.2 ^a^	0.8 ± 0.1
Spleen	7 ± 1 ^b^	5 ± 2	3.0 ± 0.6 ^a^	8 ± 1
Stomach	3.5 ± 0.5 ^a^	1.9 ± 0.1 ^c^	5 ± 2	2.5 ± 0.2
Small intestine	2.6 ± 0.5 ^a^	0.7 ± 0.1 ^c^	4 ± 2 ^a^	1.0 ± 0.1
Kidney	0.3 ± 0.1 ^a,b^	0.008 ± 0.001	0.512 ± 0.002 ^a^	0.008 ± 0.001
Tumor	15 ± 4 ^b^	18 ± 3	8 ± 3 ^a^	23 ± 4
Muscle	3 ± 2 ^a^	11 ± 3 ^c^	1.1 ± 0.4 ^a^	18 ± 3
Bone	2.0 ± 0.2 ^a,b^	43 ± 4 ^c^	1.6 ± 0.2 ^a^	88 ± 9

Differences were significant (*p* < 0.05) between: ^a^ the uptake of (HE)_3_-Z_HER3:08698_-DOTAGA labeled with [^125^I]I-PIB and [^111^In]In at the same time point (paired *t*-test); ^b^ the uptake of [^125^I]I-PIB-(HE)_3_-Z_HER3:08698_-DOTAGA at 4 and 24 h (unpaired *t*-test); ^c^ the uptake of [^111^In]In-(HE)_3_-Z_HER3:08698_-DOTAGA at 4 and 24 h (unpaired *t*-test).

## Abbreviations

ADAPT	Albumin-Binding Domain Derived Affinity Protein
BSA	Bovine Serum Albumin
CT	Computed Tomography
DARPin	Designed Ankyrin Repeat Protein
EDTA	Ethylenediaminetetraacetic Acid
EGFR	Epidermal Growth Factor Receptor
HER2	Human Epidermal Growth Factor Receptor 2
HER3	Human Epidermal Growth Factor Receptor 3
iTLC	Instant Thin Layer Chromatography
NODAGA	1-(1,3-carboxypropyl)-4,7-carboxymethyl-1,4,7-triazacyclononane
PBS	Phosphate-Buffered Saline
RGB SPECT	Red, Green and Blue Single Photon Emission Computed Tomography
